# Supplementary material for: Caregivers’ practices and factors associated with malaria vaccine uptake among under-five children in the Tiko Health District, Cameroon: A community based cross-sectional study
Source: PLOS Glob Public Health. 2025 Oct 21;5(10):e0004695. doi: 10.1371/journal.pgph.0004695 (PMC12539728; doi:10.1371/journal.pgph.0004695)
Supplement: Attachment — Submitted filename: PLOS ONE global health.docx [file pgph.0004695.s002.docx]

prevalence is taken 50% for sample size - is there no other estimates from the locality? or is the number reverse adjusted to match the sample size?

The standard vaccine coverage is Cluster survey (210= 7*30) why this was not considered ?

How did you rule out Cognitive impairment ? was it for the caregiver or the child ?

A majority 249 (60.7%) were married? is this significant information ?

ever attacked with malaria - can be modified as ever diagnosed or infected

Systematic sampling was used to select eligible households within each health area ? when your inclusion was under 5 , how could you use systematic sampling ? please define .

How were the variables selected for logistic regression? reference for categorical variable is missing? steps and number of iterations in logistic regression is not clear .

proportions can be supported with 95% CI

Confounders - important confounders if any ?

overall practices of caregivers - some score is used and its details ae missing in methods ?

the adverse event with vaccine plays important role in vaccine uptake - AEFI detailing if any can be added to substantiate the article title ?

The data looks good needs more focused presentation- Under 5 are the intervention group which is mentioned in title - rest all is from the care givers

its the caregivers perception and priority then the vaccine coverage as such . title , results and discussion needs to be aligned for the data they have >
