# Supplementary material for: Caregivers’ practices and factors associated with malaria vaccine uptake among under-five children in the Tiko Health District, Cameroon: A community based cross-sectional study
Source: PLOS Glob Public Health. 2025 Oct 21;5(10):e0004695. doi: 10.1371/journal.pgph.0004695 (PMC12539728; doi:10.1371/journal.pgph.0004695)
Supplement: Attachment — Submitted filename: Response to reviewers.docx [file pgph.0004695.s004.docx]

**Reviewer #1 Comments and Author Responses**

**Reviewer Comment 1:**

*"The manuscript is fairly presented, with the quality of the data, it can be further improvised. The title, method and conclusion need to be aligned to make the work more acceptable."*

**Author Response:**
Thank you for this insightful feedback. We have revised the **title** to more clearly reflect the methodology and objectives of the study. We also carefully reviewed and aligned the **methods and conclusion** sections to ensure consistency with the research aim and results. Specifically, we clarified the study design, sampling approach, and statistical procedures under the Methods section, and ensured the Conclusion reflects the findings presented in the Results. The title has been adjusted to **Caregivers’ practices and factors associated with malaria vaccine uptake among under-five children in the Tiko Health District, Cameroon: a community based cross-sectional study.**

**Reviewer Comment 2:**

*"The statistical analysis done needs to be defined properly – especially the regression – detailing the selection of variables, how the confounders were adjusted, and the reference in categorical variables."*

**Author Response:**
We appreciate your suggestion. We have now revised the **Data Analysis** section to include detailed information on the regression process:

- **Variable Selection**: All variables with a p-value < 0.20 in the bivariate analysis were included in the multivariable logistic regression model.
- **Confounder Adjustment**: Multivariable logistic regression was used to control for potential confounding by including all relevant covariates simultaneously in the model.
- **Reference Categories**: For each categorical variable included in the model, the reference category has been clearly indicated in the revised tables and described in the text (e.g., “No education” was used as the reference group for maternal education).

We also improved the presentation of the regression results by including odds ratios (OR), 95% confidence intervals (CI), and p-values for each predictor.

**Reviewer Comment 3:**

*"The intervention group and the conclusion need to be defined properly."*

**Author Response:**
Thank you for pointing this out. We have clarified the term "intervention group" throughout the manuscript. Since this study focused on assessing the uptake and determinants of malaria vaccination (and did not involve an active intervention), we have removed or rephrased all references that may have implied a randomized controlled trial or intervention study design.

We have also revised the **Conclusion** to ensure it is based solely on the study findings, avoiding any implication of causality or intervention effects. The conclusion now clearly highlights the key factors associated with malaria vaccine uptake and suggests recommendations for targeted health education based on the observed determinants.

**Response to Reviewer #2**

We sincerely thank Reviewer #2 for the insightful and constructive feedback, which has significantly improved the quality of our manuscript. We have carefully addressed all comments and revised the manuscript accordingly. Below is a point-by-point response:

**General Comment:**

- We acknowledge the grammatical issues and have revised the manuscript thoroughly for clarity, grammar, and tone to meet the journal’s standards. We used professional editing tools and sought peer review to ensure quality improvement.

**Specific Comments:**

**1. Line 65–67:**
**Comment:** Please provide the recent numbers from WHO World Malaria Report 2024.
**Response:** We have updated the statistics with the most recent figures from the WHO World Malaria Report 2024 and provided an appropriate citation.

**2. Line 111:**
**Comment:** Sample size formula has mentioned *d²* in the denominator; however, *E²* is abbreviated.
**Response:** Thank you for noting the inconsistency. We have corrected the abbreviation to maintain consistency between the formula and the explanation, using *e²* throughout.

**3. Lines 118–123:**
**Comment:** Provide detailed sampling methodology.
**Response:** We have revised the Methods section to include the following:

- Six health areas (Tiko Town, Holforth, Mutengene, Misselele, Kange, and Likomba) were selected from the eight using the simple random sampling method via balloting.
- The proportional-to-population size (PPS) method was used. The proportions and number of participants finally selected per health area are clearly indicated on a table
- **4. Line 162:**
  **Comment:** “A vast majority 329 (80.2%) of the caregivers were direct parents of the under……children?”
  **Response:** Sentence has been corrected to: “A vast majority (329; 80.2%) of the caregivers were the biological parents of the under-five children.”

**5. Table 1:**

- **Household income:** Currency has been specified as Central African CFA francs (XAF).
- **Smoking status:** Changed to “Smoker/Non-smoker.”
- **Child ever attacked with malaria:** Rephrased as “History of malaria episodes in the child.”

**6. Lines 168–170:**
**Comment:** Sentence incomplete and grammatically incorrect.
**Response:** We have revised and completed the sentence for clarity and grammar.

**7. Table 2:**

- **“Ensures that child complete recommended vaccine doses”** changed to “Compliance with recommended vaccine doses by the child.”
- **“You actively participate…”** revised to: “Do you actively participate in community vaccination programs that offer the malaria vaccine to children?”
- **“I seek medical advice…”** rephrased as: “Seeks medical advice in case of adverse symptoms after vaccination.”

**8. Figure 1:**
**Comment:** Mention malaria vaccine instead of just “vaccination.”
**Response:** Updated figure caption and axis labels to specify “malaria vaccination.”

**9. Lines 191–193:**
**Comment:** Grammar issue.
**Response:** Sentence has been revised for grammatical correctness and clarity.

**10. Table 3:**

- “COR” now written in full as “Crude Odds Ratio (COR)” and defined in the table footnotes.
- “No vaccination” column removed to avoid redundancy.
- Entries revised:
  - “Drink alcohol” changed to “Alcohol consumption.”
  - “Child has suffered from malaria before” changed to “History of malaria in the child.”
  - “Go for general vaccination” rephrased as “Child undergoes standard immunization.”

**11. Lines 206, 208, and thereafter:**
**Comment:** aOR mentioned, but table shows COR; clarify confounders.
**Response:** We have now included both COR (Table 3) and aOR (Table 4) in clearly separated tables. In the Methods section, we clarified that variables significant at *p* < 0.2 in bivariate analysis were included in the multivariable logistic regression model to estimate aORs, adjusting for potential confounders including caregiver education level, household income, vaccine knowledge score, and distance to health facility.

**12. Line 211:**
**Comment:** XAF – abbreviate.
**Response:** Defined XAF as Central African CFA franc at first mention and included abbreviation thereafter.

**13. Lines 218–220:**
**Comment:** Sentence incomplete.
**Response:** The sentence has been completed and revised for clarity.

**14. Table 4:**

- “COR” replaced with “Adjusted Odds Ratio (aOR)” and footnoted.
- “No vaccination” column removed.

**15. Lines 230–235:**
**Comment:** Grammar issues and need for clarity.
**Response:** These lines have been rephrased for improved clarity and grammatical correctness.

We appreciate the reviewer’s efforts in helping us improve the manuscript and believe the revisions now align well with the journal’s standards.
