# Supplementary material for: Caregivers’ practices and factors associated with malaria vaccine uptake among under-five children in the Tiko Health District, Cameroon: A community based cross-sectional study
Source: PLOS Glob Public Health. 2025 Oct 21;5(10):e0004695. doi: 10.1371/journal.pgph.0004695 (PMC12539728; doi:10.1371/journal.pgph.0004695)
Supplement: Attachment — Submitted filename: Response_to_Reviewers_auresp_3.docx [file pgph.0004695.s006.docx]

**Response to Reviewers**

**Manuscript ID:** PGPH-D-25-01162R2
**Title:** Caregivers’ practices and factors associated with malaria vaccine uptake among under-five children in the Tiko Health District, Cameroon: a community-based cross-sectional study

We thank the Academic Editor for the helpful feedback and guidance. We have carefully revised the manuscript according to all comments. Below are point-by-point responses:

1. Comment: “‘Questionnaires’ is still used in the abstract, line 344 and elsewhere.”
   **Response:** We corrected “questionnaires” to “questionnaire” throughout the abstract and manuscript.
2. Comment: “Lines 275 to 277 may be added to the first paragraph of the discussion.”
   **Response:** The specified lines have been moved to the end of the first discussion paragraph.
3. Comment: “Check the font type for lines 186 to 188.”
   **Response:** Font inconsistency corrected.
4. Comment: “Line 204: check punctuation (full stop).”
   **Response:** Punctuation corrected.
5. Comment: “The amounts in US dollars in the tables may be rounded off to the nearest whole number.”
   **Response:** All amounts in US dollars in the tables have been rounded to the nearest whole number.
6. Comment: “Line 293 to 298: No reference to existing literature is made. Indeed, it is a shallow discussion paragraph.”
   **Response:** We revised this section and added relevant references to strengthen the discussion.
7. Comment: “Very long discussion paragraph from line 299 to 332.”
   **Response:** This paragraph was split into three shorter paragraphs for better clarity.
8. Comment: “Line 333 to 340: reference to existing literature is needed for all discussion paragraphs.”
   **Response:** Additional references were added to support statements in these paragraphs.
9. Comment: “Conclusion may be a single paragraph.”
   **Response:** The conclusion has been condensed into a single, concise paragraph.

We believe these revisions address all comments and improve the clarity and quality of the manuscript. We sincerely thank the Editor for the constructive feedback and consideration of our work.
