# Supplementary material for: Caregivers’ practices and factors associated with malaria vaccine uptake among under-five children in the Tiko Health District, Cameroon: A community based cross-sectional study
Source: PLOS Glob Public Health. 2025 Oct 21;5(10):e0004695. doi: 10.1371/journal.pgph.0004695 (PMC12539728; doi:10.1371/journal.pgph.0004695)
Supplement: Attachment — Submitted filename: Response to Editor and Reviewer Comments.docx [file pgph.0004695.s005.docx]

**Response to Editor and Reviewer Comments**
Manuscript ID: PGPH-D-25-01162R1
Title: *Caregivers’ practices and factors associated with malaria vaccine uptake among under-five children in the Tiko Health District, Cameroon: a community-based cross-sectional study*

Dear Dr. Musoke,

We sincerely thank you and the reviewers for the thoughtful and constructive feedback provided on our revised manuscript. We appreciate the time and effort taken to improve the quality of our work. We have carefully revised the manuscript in line with the editorial comments. Below, we provide a detailed, point-by-point response outlining the changes made.

**Editorial Comments**

1. **Title not consistent with the aim of the study in the abstract**
   - *Response:* Thank you for this observation. To ensure alignment, we adjusted the aim in the abstract to read:
     *“This study assessed caregivers’ practices and factors associated with malaria vaccine uptake among under-five children in the Tiko Health District.”*
2. **Line 41: Structured questionnaires or rather a restructured questionnaire?**
   - *Response:* We have corrected the wording to “structured questionnaire” to accurately reflect the tool used.
3. **Currency should be converted to USD throughout the manuscript**
   - *Response:* All monetary values have been converted to USD with the equivalent exchange rate stated. A sentence has been included in the methodology section (line 173 to 175) to indicate this conversion. The sentence reads “All costs were originally recorded in Central African Francs (XAF) and converted to United States Dollars (USD) using the prevailing exchange rate at the time of the study (1 USD = XXX XAF)”
4. **Line 92: … study aimed to …**
   - *Response:* This has been corrected to improve grammatical clarity.
5. **Lines 119–120: Why sampling technique twice?**
   - *Response:* We have removed the redundancy and streamlined the description of the sampling procedure.
6. **Background and level of training of the Research Assistants**
   - *Response:* Response: Thank you for this valuable comment. We have now added details on the research assistants’ background and training in the Methods section. Specifically, we clarified that the research assistants were public health graduates with prior experience in survey data collection. They underwent a two-day training that covered the study objectives, ethical considerations, informed consent, confidentiality, interview techniques, and piloting of the questionnaire. This ensures methodological rigor and data quality (Line 158-163).
7. **More details on training of research assistants (content and number of days)**
   - *Response:* We now specify that research assistants underwent a two-day training, which included familiarization with the study objectives, ethical procedures, interview techniques, and piloting of the questionnaire.
8. **How was the questionnaire designed? Was reference made to any existing literature? If so, cite.**
   - *Response:* We have revised the methods to clarify that the questionnaire was developed after reviewing relevant literature on malaria vaccination and immunization studies in sub-Saharan Africa. Appropriate references have been cited(Line 152-158).
9. **How were caregivers defined? Did they include mothers?**
   - *Response:* We now provide a definition: caregivers were defined as adults (≥18 years) primarily responsible for the day-to-day care of the child, including mothers, fathers, or guardians. This has been added to the methods section under study population (Line 108-111).
10. **Figure 2 may not be necessary**

- *Response:* Figure 2 has been removed as suggested.

11. **Statistically significant results should be indicated in the tables**

- *Response:* We have indicated statistically significant results in all tables with appropriate symbols and footnotes.

12. **First paragraph of the discussion should provide an overview of findings and significance**

- *Response:* We have restructured the discussion. The first paragraph now provides a concise overview of the key findings and their relevance to public health, followed by a more detailed interpretation in subsequent paragraphs.

13. **Separate paragraphs for various key findings in discussion**

- *Response:* The discussion has been divided into separate paragraphs addressing different key findings for clarity and flow.

14. **Study limitations (and strengths) needed at the end of discussion**

- *Response:* A new subsection has been added at the end of the discussion, highlighting both the strengths and limitations of the study.

15. **Subsection of ‘recommendations’ not expected in manuscript**

- *Response:* The recommendations subsection has been removed. Relevant recommendations have been incorporated into the concluding sentences of the discussion.

16. **No need to have statistics in the conclusion**

- *Response:* All statistics have been removed from the conclusion. The conclusion now provides a concise summary of the findings and their implications without numerical details.

**Reviewer #1**

We appreciate Reviewer #1’s positive feedback, stating that all prior comments were addressed and the manuscript is now much improved.

We trust that the revisions made have addressed the editor’s and reviewer’s concerns satisfactorily. We hope that the manuscript is now acceptable for publication in *PLOS Global Public Health*.

Sincerely,
Dr. Ukah Chrisantus Eweh (on behalf of all co-authors)
